# Comparison of Balloon Pulmonary Angioplasty and Pulmonary Vasodilators for Inoperable Chronic Thromboembolic Pulmonary Hypertension: A Systematic Review and Meta-Analysis

**DOI:** 10.1038/s41598-020-65697-4

**Published:** 2020-06-01

**Authors:** Rajat Kalra, Sue Duval, Thenappan Thenappan, Ganesh Raveendran, Marc Pritzker, Sasha Z. Prisco, Kurt W. Prins

**Affiliations:** Cardiovascular Division, University of Minnesota, Minneapolis, MN USA

**Keywords:** Cardiology, Interventional cardiology

## Abstract

Treatment options for chronic thromboembolic pulmonary hypertension (CTEPH) that is not amenable to thromboendarterectomy or is recurrent/persistent after thromboendarterectomy (inoperable CTEPH) include pulmonary vasodilators or balloon pulmonary angioplasty (BPA). We compared efficacy and safety outcomes of BPA with or without pulmonary vasodilators to pulmonary vasodilator therapy alone in patients with inoperable CTEPH. Observational and randomized trial data reporting outcomes for >5 patients with inoperable CTEPH were sought. Single-arm random effects meta-analyses were performed. The primary outcome was change in six-minute walk distance (6MWD). Secondary outcomes included safety; World Health Organization functional class (WHO FC); and change in mean pulmonary arterial pressure (mPAP), pulmonary vascular resistance (PVR), and cardiac index. Thirty-four studies with 1604 patients were eligible for analyses. Both treatments resulted in significant improvement in 6MWD (71.0 meters, 95% CI: 47.4–94.5 meters with BPA versus 47.8 meters, 95% CI: 34.5–61.2 meters with pulmonary vasodilators), PVR [−3.1 Wood Units (WU), 95% CI: −4.9 to −1.4 WU versus −1.6 WU, 95% CI: −2.4 to −0.8 WU] and mPAP (−14.8 mmHg, 95% CI: −18.2 to −11.5 mmHg versus −4.9 mmHg, 95% CI: −6.9 to −2.8 mmHg). Cardiac index was similar and most patients were WHO FC II and III after their respective interventions. More complications occurred in the BPA arm. In conclusion, BPA and pulmonary vasodilators both improve 6MWD and hemodynamics in patients with inoperable CTEPH. While BPA may offer greater functional and hemodynamic improvements, this technique carries the accompanying risks of an invasive procedure.

## Introduction

Chronic thromboembolic pulmonary hypertension (CTEPH) is characterized by macrovascular obstruction due to thromboemboli with an accompanying small vessel pulmonary arteriopathy^1^. Pulmonary thromboendarterectomy is the gold standard treatment for CTEPH with numerous centers of excellence worldwide^2,3^. However, over 40% of CTEPH patients are ineligible for pulmonary thromboendarterectomy, due to a combination of technical inaccessibility of thromboemboli, poor surgical candidacy, patient choice, or recurrent/persistent pulmonary hypertension after the operation^4,5^.

CTEPH patients who are not amenable to thromboendarterectomy, develop CTEPH recurrence after thromboendarterectomy, or have persistent CTEPH despite thromboendarterectomy (inoperable CTEPH) have worse outcomes than patients who successfully undergo operative intervention^6,7^. In view of this, new treatments have emerged for this patient population. Balloon pulmonary angioplasty (BPA) is a percutaneous approach that employs sequential pulmonary artery angioplasty to relieve the macrovascular obstruction associated with CTEPH^6^. This approach improves exercise capacity and hemodynamics^8^. Furthermore, pulmonary vasodilators are used to treat the accompanying small vessel arteriopathy in CTEPH^9^. Riociguat, a soluble guanylate cyclase activator, improves exercise capacity and hemodynamics in inoperable CTEPH^10^. It is therefore approved by the Food and Drug Administration to medically treat inoperable CTEPH. Macitentan and subcutaneous treprostinil also improved in exercise capacity in phase II clinical trials in inoperable CTEPH patients^11,12^. However, there are limited data comparing the efficacy and safety of medical therapies to BPA in the inoperable CTEPH population.

We sought to compare the efficacy and safety outcomes of BPA and pulmonary vasodilator therapy in patients with inoperable CTEPH. We hypothesized that BPA with or without pulmonary vasodilator therapy would provide superior improvements in exercise capacity and hemodynamics, with similar safety outcomes when compared to pulmonary vasodilators alone. Here, we present the results of a systematic review and single-arm meta-analyses that investigate the aforementioned hypotheses.

## Methods

The study protocol is detailed in Supplemental Appendix 1.

### Search strategy

The SCOPUS database was searched from inception (1945) to August 2019 for eligible studies using a prespecified term list. SCOPUS catalogues MEDLINE, Embase, Compendex, the World Textile index, Fluidex, Geobase, and Biobase^13^. The full search strategy is detailed in Supplemental Appendix 2.

### Study characteristics

Patients with CTEPH were defined as being inoperable if they were deemed by study authors to have distal disease that was not amenable to surgery, unacceptable perioperative risk, recurrence of CTEPH after surgery, or their CTEPH was persistent after surgery^7^. Studies were included if they detailed outcomes for five or more patients. Observational and randomized trial data were both sought. Studies reporting outcomes for patients receiving both BPA and pulmonary vasodilators were included in the BPA arm. Conference abstracts were eligible if they reported the primary outcome. Foreign language manuscripts that did not have an English translation and unpublished studies were excluded. Where multiple studies reported outcomes on the same patient cohort, the study report with the longest person-year follow-up and most complete outcome reporting was included.

### Outcome measures

The primary outcome was change in six-minute walk distance (6MWD). The secondary outcomes included World Health Organization (WHO)/New York Heart Association functional status, change in mean pulmonary arterial pressure (mPAP), change in pulmonary vascular resistance (PVR), change in cardiac index, and safety outcomes. The safety outcomes were divided into reperfusion pulmonary edema, wire injuries, and all-cause mortality during follow-up for the BPA arm. For the medical arm, the safety outcomes were divided into serious adverse events and all-cause mortality during follow-up. Adverse events were defined as serious if they necessitated admission to the hospital or prolonged existing hospitalization, led to an unplanned procedure, led to discontinuation of therapy, or were described as being life-threatening or causing severe disability. Any other events that the study authors defined as serious were also classified as serious adverse events. The outcome definitions are also outlined in Supplemental Appendix 3.

### Data extraction

A single investigator performed data extraction (R.K.) with random and blinded verification for consistency in data extraction by two other authors (K.P. and T.T.). Study quality was assessed via the Newcastle-Ottawa scale for observational studies^14^ and the Cochrane Risk of Bias Tool for randomized controlled trials^15^. All disagreements in study design and data extraction were resolved via mutual consensus.

### Data synthesis and statistical analyses

Categorical variables were represented as counts with proportions. Continuous variables were represented as means and 95% confidence intervals (95% CI). If authors reported medians and interquartile ranges, means and standard deviations were calculated. Standard deviations were estimated without knowledge of the correlation between pre- and post-treatment values where authors did not provide standard deviations. These estimates likely constituted an over-estimate of the standard deviation and produced more conservative results. Estimates of variance were digitally extracted where they were not reported in text by study authors^16^. The methods used to transform study-level data to a consistent form are outlined in full in Supplemental Appendix 4. Meta-analysis of proportions was used to summarize categorical baseline characteristics and outcomes. Where a proportion of 0 or 1 was noted, a continuity correction of 0.5 was applied. Meta-analysis of continuous variables was done with means and standard errors. Random effects models with inverse variance weighting were used to provide the most conservative effect estimates and 95% confidence intervals, as we expected a priori to see considerable variability between studies. I^2^ values were used to assess for heterogeneity^17^. Two-tailed *p*-values were used for hypothesis testing and the significance level was set at 0.05. Publication bias was assessed using Egger’s regression^18^ and the Trim and Fill method to impute missing studies^19^. This meta-analysis was reported in accordance with the MOOSE Checklist for Meta-Analyses of Observational Studies^20^.

All statistical analyses were conducted in R and R Studio version 1.1.463 (R Foundation for Statistical Computing, Vienna, Austria)^21^ and Stata MP, version 15.2 (College Station, TX, U.S.A.).

## Results

**Figure 1:**
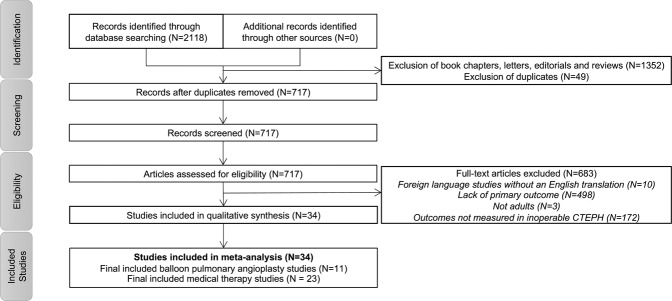
Flow Diagram for Study Selection.

A total of 34 studies detailing outcomes for 1604 patients were identified for inclusion in the analyses (Fig. 1). Of these, 11 studies presented outcomes for 755 patients with inoperable CTEPH treated with BPA (Table 1)^8,22–31^. Twenty-three studies reported outcomes in 849 patients with inoperable CTEPH treated with pulmonary vasodilators (Table 1)^10–12,32–51^. The MOOSE checklist is detailed in Supplemental Appendix 5. The Newcastle-Ottawa and Cochrane Risk of Bias quality assessments of the included studies is detailed in Supplemental Appendix 6. The overall quality of the observational studies was modest to high with scores ranging from 4–8. There were concerns about bias in only one of the six included randomized trials.

**Table 1:** Baseline Characteristics of Patients in Included Studies.

Study (Design)	Number of Patients (Single or Multiple Centers)		Male Patients n (%)		Mean Age (Years)		Person Years Follow-Up		WHO FC I n (%)		WHO FC II n (%)		WHO FC III n (%)		WHO FC IV n (%)	Mean Number of Catheterization Procedures			Proportion on Vasodilator Therapy		Mean Baseline 6MWD (m)		Mean Baseline mPAP (mmHg)		Mean Baseline CI (L/min/m^2^)		Mean Baseline PVR (Wood units)
**Balloon Pulmonary Angioplasty**																											
Feinstein *et al*., 2001 (Observational)	18 (Single)		NR		52		51.3		NR		NR		NR		NR	2.7			NR		191.1		42.0		2.0		22.0
Roik *et al*., 2016 (Observational)	10 (Single)		4 (40%)		81		15.4		0		0		7 (70%)		3 (30%)	3.9			6 (60%)		210.0		41.5		2.3		8.9
Moriyama *et al*., 2017 (Observational)	53 (Single)		13 (24.5%)		63		NR		0		11 (21%)		36 (68%)		6 (11%)	6.0			NR		351.4		37.2		2.2		8.4
Ogawa *et al*., 2017 (Observational)	308 (Multiple)		62 (20.1%)		62		364.0		0		56* (18%)		192* (62%)		43* (14%)	8.3			222 (71%)		318.1		43.2		2.6		10.7
Yamasaki *et al*., 2017 (Observational)	20 (Single)		4 (20.0%)		62		NR		0		2 (10%)		17 (85%)		1 (5%)	2.7			20 (100%)		391.0		42.6		3.1		8.0
Kriechbaum *et al*., 2018 (Observational)	51 (Single)		23 (45.1%)		63		25.5		0		2 (4%)		31 (61%)		18 (35%)	5.2			29 (57%)		367.2		39.5		NR		6.5
Kurzyna *et al*., 2018 (Observational)	31 (Single)		NR		NR		32.3		0		1 (3%)		23 (74%)		7 (23%)	NR			NR		306.0		50.7		2.3		10.3
Kwon *et al*., 2018 (Observational)	15 (Single)		8 (53.3%)		53		11.3		0		6 (40%)		5 (33%)		4 (27%)	3.5			9 (60%)		387.0		NR		2.9		7.6
Velazquez *et al*., 2018 (Observational)	46 (Single)		32 (69.6)		14 (30%)		54.8		0		5/43 (12%)		30/43 (70%)		8/43 (19%)	NR			46 (100%)		394.5		49.5		2.3		10.1
**Balloon Pulmonary Angioplasty (continued)**																											
Yamagata *et al*., 2018 (Observational)	19 (Single)		3 (15.8%)		68		NR		0		3 (16%)		16 (84%)		0	3.2			NR		308.1		40.1		NR		7.5
Brenot *et al*., 2019 (Observational)	184 Multiple)		94 (51.1%)		63		93.5		2 (1%)		64 (35%)		109 (59%)		9 (5%)	5.4			57%		396		44.1		2.7		7.6
**Pulmonary Vasodilators**																											
Ghofrani *et al*., 2003 (Observational)		12 (Single)		7 (58%)		NR		6.5		NR		NR		NR			NR	Sildenafil PO (50 mg TID/ 6 months)		312		NR		2.0		NR	
Scelsi *et al*., 2004 (Observational)		11 (Single)		6 (55%)		50		11.4		0		0		11 (100%)			0	Epoprostenol IV (12.7 + 6.8 ng/kg/min/ 12 months)		253		44.0		NR		12.0	
Bonderman *et al*., 2005 (Observational)		16 (Single)		7 (44%)		70		8.0		0		10 (63%)		4 (25%)			2 (13%)	Bosentan PO (125 mg BID/ 6 months)		299		56.0		1.9		8.9	
Hoeper *et al*., 2005 (Observational)		18 (Multiple)		11 (61%)		60		4.5		0		2 (11%)		14 (88%)			2 (11%)	Bosentan PO (125 mg BID/ 3 months)		340		47.0		2.1		11.4	
Hughes *et al*., 2006 (Observational)		47 (Multiple)		20 (43%)		56		53.3		0		10 (21%)		32 (68%)			5 (11%)	Bosentan PO (62.5 mg BID/ 12 months)		291		NR		NR		NR	
Vizza *et al*., 2006 (Observational)		8 (Single)		1 (13%)		44		4.0		0		3 (38%)		4 (50%)			1 (13%)	Beraprost PO (275 + 47 μg/ 6 months)		313		48.0		2.4		11.0	
Cabrol *et al*., 2007 (Observational)		27 (Single)		13 (48%)		51		27.8		0		0		20 (74%)			7 (26%)	Epoprostenol IV (16 + 2.8 ng/kg/min/ 20 months)		265		52.0		2.1		NR	
**Pulmonary Vasodilators (continued)**																											
Reichenberger *et al*., 2007 (Observational)		104 (Single)		45 (43%)		62		104.0		0		8 (8%)		76 (73%)			20 (19%)	Sildenafil PO (50 mg TID/ 12 months)		310		44.2		NR		10.8	
Segovia Cubero *et al*., 2007 (Observational)		6 (Single)		1 (17%)		60		7.5		NR		NR		NR			NR	Bosentan PO (125 mg BID/ 15 months)		230		55.0		1.9		12.6	
Seyfarth *et al*., 2007 (Observational)		12 (Single)		5 (42%)		57		24.0		0		0		12 (100%)			0	Bosentan PO (125 mg BID/ 24 months)		319		45.8		2.2		12.6	
Skoro-Sajer *et al*., 2007 (Observational)		25 (Single)		9 (36%)		60		50.0		0		0		11 (44%)			14 (56%)	Treprostinil IV (28 + 10 ng/kg/min 19 months)		260		41.0		NR		11.6	
Jais *et al*., 2008 (RCT)		77 (Multiple)		22 (29%)		63		25.7		0		22 (29%)		51 (66%)			3 (4%)	Bosentan PO (125 mg BID/ 4 months)		340		NR		NR		9.7	
Rossi *et al*., 2008 (Observational)		9 (Single)		2 (22%)		67		4.5		0		0		8 (89%)			1 (11%)	Sildenafil PO (100 mg TID/ 6 months)		244		NR		2.30		14.4	
Suntharalingam *et al*., 2008 (RCT)		9 (Single)		2 (22%)		49		2.3		0		3 (33%)		6 (67%)			0	Sildenafil PO (40 mg TID/ 3 months)		331		45.0		NR		10.1	
Post *et al*., 2009 (Observational)		18 (Single)		11 (61%)		63		49.5		0		15 (83%)		3 (17%)			0	Bosentan PO (125 mg BID/ 31 months)		405		49.9		2.20		7.8	
Vassallo *et al*., 2009 (Observational)		17 (Multiple)		2 (12%)		65		17.0		0		1 (6%)		14 (82%)			2 (12%)	Bosentan PO (125 mg BID/ 12 months)		297		49.9		2.30		NR	
Ghofrani *et al*., 2010 (Observational)		41 (Multiple)		23 (56%)		63		10.3		0		10 (24%)		31 (76%)			0	Riociguat PO (2.5 mg TID/ 3 months)		387		43.8		2.31		8.6	
**Pulmonary Vasodilators (continued)**																											
Ghofrani *et al*., 2013 (RCT)		173 (Multiple)		55 (32%)		59		57.7		3 (2%)		55 (32%)		107 (62%)			8 (5%)	Riociguat PO (2.5 mg TID/ 4 months)		342		44.0		NR		9.9	
Ghofrani *et al*., 2017 (RCT)		40 (Multiple)		14 (35%)		58		20.0		0		12 (30%)		28 (70%)			0	Macitentan PO (10 mg daily/ 4 months)		353		56.0		1.90		11.6	
Yamamoto *et al*., 2017 (Observational)		23 (Single)		3 (12%)		66		23.0		0		20 (87%)		3 (13%)			0	Riociguat (2.5 mg TID/ 12 months)		373		38		3.0		6.7	
Sadushi-Kolici *et al*., 2018 (RCT)		105 (Multiple)		56 (53%)		64		52.5		0		6 (6%)		91 (87%)			8 (8%)	Treprostinil SC (30 ng/kg/min/ 3 months)		303		47.0		2.10		10.3	
Escribano-Subias *et al*., 2019 (RCT)		15 (Multiple)		NR		NR		5.0		NR		NR		NR			NR	Ambrisentan (5-10 mg daily/ 4 months)		NR		NR		NR		NR	
Van Thor *et al*., 2019 (Observational)		36 (Multiple)		18 (50%)		65		545.2		0		16 (46%)		18 (51%)			1 (3%)	Riociguat (2.5 mg TID/ mean 28 months)		337		38.1		NR		6.1	

Legend: *: Incomplete reporting of baseline characteristics, μg: Micrograms, 6MWD: Six-Minute Walk Distance, BID: Bis in Die (Twice Daily), CI: Cardiac Index, IV: Intravenous, L/min/m^2^: Liters per minute per meters squared, m: meters, mmHg: Millimeters of mercury, mPAP: Mean Pulmonary Artery Pressure, n: number, ng/kg/min: Nanograms/kilograms/minute, NR: Not Reported, PO: Per OS (oral), PVR: Pulmonary Vascular Resistance, RCT: Randomized Controlled Trial, SC: Subcutaneous, TID: Ter in Die (Three Times Daily), WHO FC: World Health Organization Functional Class.

### Baseline characteristics for patients treated with balloon pulmonary angioplasty

The baseline characteristics of inoperable CTEPH patients who underwent BPA are outlined in Table 1. Within this cohort, the mean age was 62.8 years (95% CI: 59.9–65.6) and 41.2% of patients were male (95% CI: 7.5–74.6%). The majority of patients were WHO FC III (67.8%, 95% CI: 61.5–73.7%) or WHO FC IV (16.5%, 95% CI: 9.7–23.3%) at baseline. Amongst BPA studies, 74.3% of patients were on vasodilators at baseline (95% CI: 58.5–90.0%).

There was variable reporting of functional and hemodynamic indices. The mean baseline 6MWD was 344.8 meters (95% CI: 314.6–375.0 meters). The mean pre-procedural mPAP was 43.1 mmHg (95% CI: 40.9–45.2). The mean pre-procedural PVR was 9.2 Wood units (95% CI: 8.0–10.5 Wood units) and the mean pre-procedural cardiac index was 2.5 L/min/m^2^ (95% CI: 2.3–2.7 L/min/m^2^).

The procedural protocol for BPA varied amongst the included studies. The mean number of procedures was 4.9 (95% CI: 3.1–6.7 procedures) and the patients underwent a mean 7.1 vessel angioplasties per procedure (95% CI: 0.8–13.3 vessels).

### Baseline characteristics for patients treated with pulmonary vasodilators

The baseline characteristics of inoperable CTEPH patients who received pulmonary vasodilators are also outlined in Table 1. The mean age of the patients was 59.8 years (95% CI: 57.5–62.2 years). Patients were treated with ambrisentan, beraprost, bosentan, macitentan, riociguat, sildenafil, intravenous epoprostenol and intravenous or subcutaneous treprostinil. Amongst all 23 studies, 38.2% (95% CI: 31.8–44.6%) of the patients were male. The majority of patients were WHO FC II (24.2%, 95% CI: 15.3–39.3%) or WHO FC III (65.3%, 95% CI: 55.1–75.4%) prior to treatment with pulmonary vasodilators. Treatment duration ranged from 3–24 months (Table 1).

The mean baseline 6MWD was 316.0 meters (95% CI: 300.2–331.8 meters). The mean pre-treatment mPAP was 47.4 mmHg (95% CI: 45.6–49.2 mmHg). The mean pre-treatment PVR was 9.9 Wood units (95% CI: 9.1–10.7 Wood units). The mean pre-treatment cardiac index was 2.2 L/min/m^2^ (95% CI: 2.1–2.3 L/min/m^2^).

### Efficacy outcomes

**Figure 2:**
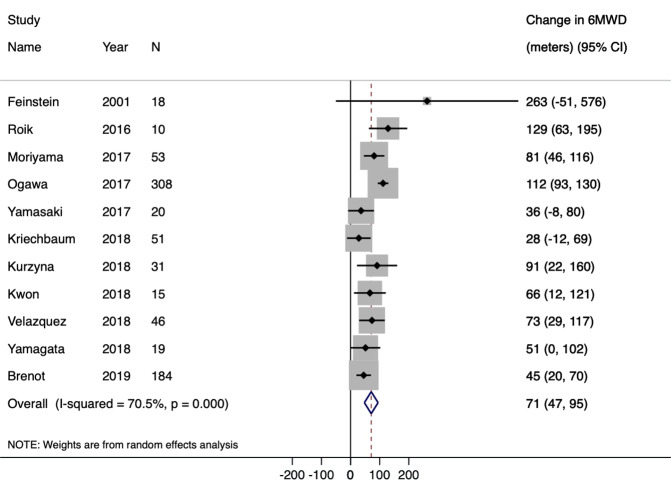
Forest Plot for Primary Outcome (Change in 6MWD) for Balloon Pulmonary Angioplasty Arm. The black diamonds and lines represent the point estimate and 95% confidence intervals, respectively. The blue diamond represents the pooled effect estimate.

The mean change in 6MWD after the last catheterization procedure in the BPA studies was 71.0 meters (95% CI: 47.4–94.5 meters) (Fig. 2 and Table 2). The mean reduction in PVR was −3.1 Wood units (95% CI: −4.9 to −1.4 Wood units). The mean reduction in mPAP was −14.8 mmHg (95% CI: −18.2 to −11.5 mmHg). The mean increase in cardiac index was 0.2 L/min/m^2^ (95% CI: 0.1–0.4 L/min/m^2^) (Table 2). After the last BPA procedure, the majority of patients were classified as WHO FC I (31.4%, 95% CI: 11.0–51.8%) or WHO FC II (50.4%, 95% CI: 32.2–68.6%).

**Table 2:** Efficacy and Safety Outcomes.

Efficacy Outcomes			
Efficacy Outcome	Number of Studies Reporting the Outcome	Mean Change (95% Confidence Interval)	Estimate of Heterogeneity (I^2^)
**Balloon Pulmonary Angioplasty**			
6-Minute Walk Distance, meters	11/11	71.0 (47.4–94.5)	70.5%
Cardiac Index, L/min/m^2^	10/11	0.2 (0.1–0.4)	60.3%
Mean Pulmonary Artery Pressure, mmHg	10/11	−14.8 (−18.2 to −11.5)	83.6%
PVR, Wood units	10/11	−3.1 (−4.9 to −1.4)	95.2%
**Pulmonary Vasodilators**			
6-Minute Walk Distance, meters	23/23	47.8 (34.5–61.2)	94.4%
Cardiac Index, L/min/m^2^	13/23	0.3 (0.2–0.4)	46.1%
Mean Pulmonary Artery Pressure, mmHg	11/23	−4.9 (−6.9 to −2.8)	78.2%
PVR, Wood units	12/23	−1.6 (−2.4 to −0.8)	73.1%
**Safety Outcomes**			
**Safety Outcome**	**Number of Studies Reporting the Outcome**	**Point Estimate of Incidence (95% Confidence Interval)**	**Estimate of Heterogeneity**
**Balloon Pulmonary Angioplasty**			
All-Cause Mortality	8/11	3.4% (2.0–4.9%)	0.0%
Reperfusion Pulmonary Edema	8/11	12.9% (7.7–18.2%)	91.0%
Wire Injuries	6/11	5.3% (3.3–7.2%)	60.6%
**Pulmonary Vasodilators**			
**Outcomes**	**Number of Studies Reporting the Outcome**	**Point Estimate of Incidence (95% Confidence Interval)**	**Estimate of Heterogeneity**
All-Cause Mortality	20/23	1.3% (0.1–2.3%)	16.0%
Serious Adverse Events	19/23	9.6% (4.7–14.6%)	90.2%

**Figure 3:**
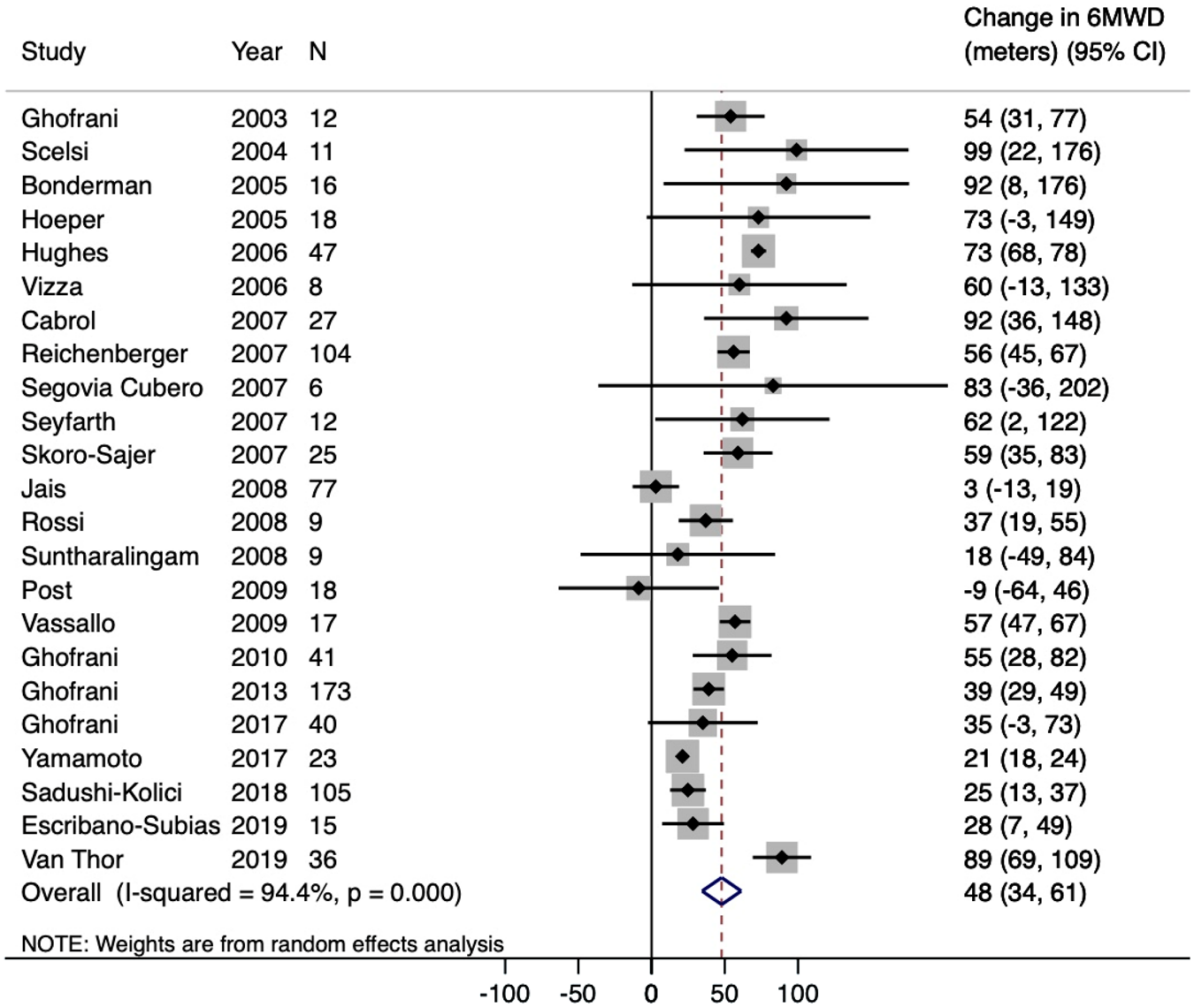
Forest Plot for Primary Outcome (Change in 6MWD) for Pulmonary Vasodilator Arm. The black diamonds and lines represent the point estimate and 95% confidence intervals, respectively. The blue diamond represents the pooled effect estimate. p = 0.32.

The mean change in 6MWD in the pulmonary vasodilator studies was 47.8 meters (95% CI: 34.5–61.2 meters) (Fig. 3 and Table 2). The mean reduction in PVR was −1.6 Wood units (95% CI: −2.4 to −0.8 Wood units). The mean reduction in mPAP was −4.9 mmHg (95% CI: −6.9 to −2.8 mmHg). The mean increase in cardiac index was 0.3 L/min/m^2^ (95% CI: 0.2–0.4 L/min/m^2^). The majority of patients were classified as WHO FC II (34.9%, 95% CI: 23.2–46.7%) or WHO FC III (51.9%, 95% CI: 36.7–66.7%) after treatment.

### Safety outcomes

In the balloon pulmonary angioplasty arm, the mean incidence of reperfusion edema was 12.9% (95% CI: 7.7–18.2%). The estimated incidence of wire injuries was 5.3% (95% CI: 3.3–7.2%). The incidence of all-cause mortality was estimated to be 3.4% (95% CI: 2.0–4.9%) over 648.1 person years follow-up (Table 2).

In the pulmonary vasodilators arm, the estimated incidence of serious adverse events was 9.6% (95% CI: 4.7–14.6%). The incidence of all-cause mortality was estimated to be 1.3% (95% CI: 0.1–2.3%) over 1136.5 person years follow-up (Table 2).

### Sensitivity analyses

**Figure 4:**
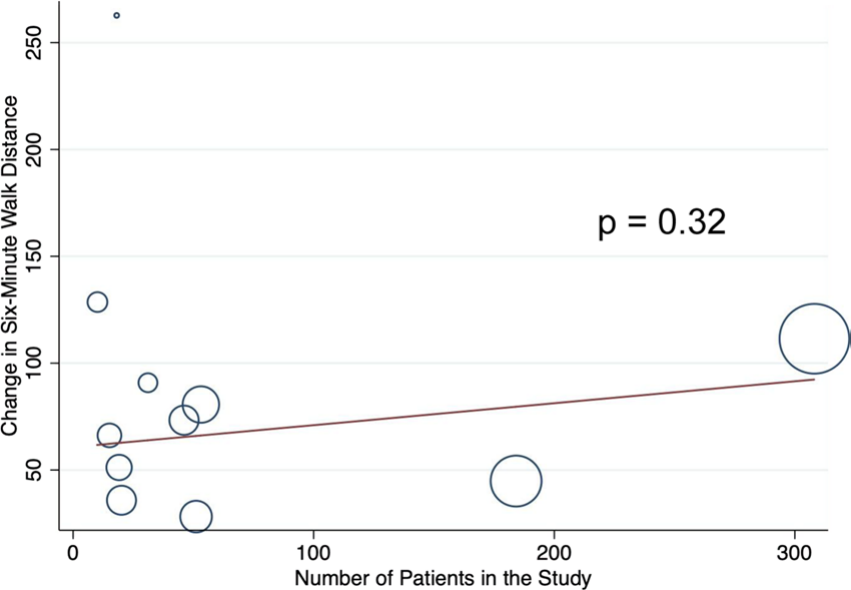
Meta-Regression of Change in Six-Minute Walk Distance by Procedural Volume in Balloon Pulmonary Angioplasty Studies.

There was significant heterogeneity in the change in 6MWD in the BPA arm (I^2^ = 70.5%). In order to evaluate heterogeneity, meta-regression was performed to evaluate the effect of procedural volume on change in 6MWD after BPA. The number of procedures reported by the authors was used as a study-level covariate. There were no differences in change in 6MWD by procedural volume (p = 0.32, Fig. 4).

**Figure 5:**
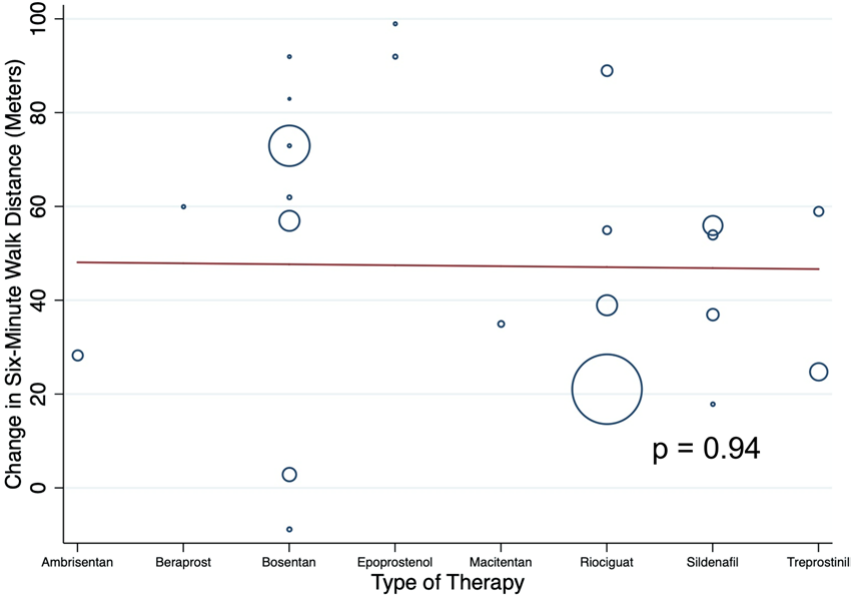
Meta-Regression of Change in Six-Minute Walk Distance by Type of Therapy in Pulmonary Vasodilator Studies.

There was also significant heterogeneity in change in 6MWD in the pulmonary vasodilators arm (I^2^ = 94.4%). Meta-regression analyses were performed to evaluate whether the type of pulmonary vasodilator affected change in 6MWD. There were no differences in change in 6MWD by the type of pulmonary vasodilator (p = 0.94, Fig. 5).

Given that riociguat is currently the only approved drug with a label to treat inoperable CTEPH in the United States, we also analyzed the subset of pulmonary vasodilator studies that evaluated riociguat usage^10,11,50,51^. The mean change in 6MWD in these studies was 49.4 meters (95% CI: 24.1–74.6 meters). The mean reduction in PVR was −2.0 Wood Units (95% CI: −3.6 to −0.5 Wood units). The mean reduction in mPAP was −6.9 mmHg (95% CI: −10.5 to −1.8 mmHg). The mean increase in cardiac index was 0.2 L/min/m^2^ (95% CI: 0.1–0.3 L/min/m^2^). These results were comparable to those pooling the results for all vasodilators.

### Publication bias

Publication bias was assessed for the primary outcome, change in 6MWD, in the pulmonary vasodilator arm with Egger’s regression and the Trim and Fill method. Egger’s regression did not indicate funnel plot asymmetry in the BPA or the pulmonary vasodilators arm (p = 0.61 and p = 0.21, respectively; Supplemental Figs. 1 and 2).

## Discussion

Here, we show that BPA and pulmonary vasodilators improve mean 6MWD, PVR, and mPAP in patients with inoperable or recurrent CTEPH after thromboendarterectomy. In our comparisons of 755 patients undergoing BPA and 849 patients receiving pulmonary vasodilators for inoperable CTEPH, BPA results in a greater improvement in 6MWD (71.0 meters, 95% CI: 47.4–94.5 meters versus 47.8 meters, 95% CI: 34.5–61.2 meters), reduction in PVR (−3.1 Wood Units, 95% CI: −4.9 to −1.4 Wood units versus −1.6 Wood units, 95% CI: 2.4 to −0.8 Wood units) and reduction in mPAP (−14.8 mmHg, 95% CI: −18.2 to −11.5 mmHg versus −4.9 mmHg 95% CI: −6.9 to −2.8 mmHg). Finally, BPA has more complications than medical therapy.

There are multiple potential explanations for our results. First, CTEPH occurs from the combination of large and moderate-sized vascular obstruction with a microvascular arteriopathy that emerges over time^1,52^. We hypothesize that the improvements in 6MWD, mPAP, and PVR in the BPA arm are related to the relief of the macrovascular obstruction. Relief of macrovascular obstruction is thought to mitigate disease progression and improve prognosis across the spectrum of pulmonary thromboembolic disease. This is evident in patients undergoing thromboendarterectomy for operable CTEPH^53^, but also in patients undergoing catheter-based intervention and surgical pulmonary embolectomy in the setting of acute pulmonary embolism^54,55^. This may partly explain why pulmonary thromboendarterectomy improves survival in CTEPH whereas pulmonary vasodilators do not^56^. However, pulmonary vasodilators improve functional and hemodynamic measures likely by targeting the microvascular arteriopathy in patients with inoperable CTEPH. The relative differences in the functional and hemodynamic changes between pulmonary vasodilators and BPA were of great interest to us. The smaller improvements observed with pulmonary vasodilators in comparison to BPA suggest that the large- and moderate-obstruction predominates in the pathology of CTEPH rather than the microvascular arteriopathy, which is consistent with the prior understanding of this disease^1,6,7^. Regardless, all of these explanations (particularly relating to the location and type of obstruction) are highly speculative and further translational research is required to elucidate these mechanisms.

Our work adds to the existing literature base pertaining to treatment strategies for inoperable CTEPH. Our investigation reports outcomes for the breadth of techniques used for BPA and the full gamut of pulmonary vasodilators that have been trialed for the treatment of inoperable CTEPH. Phan *et al*. compared hemodynamic and functional outcomes in patients with inoperable CTEPH in their meta-analysis. They found that BPA has a greater functional and hemodynamic improvement than with pulmonary vasodilators^57^. This is consistent with our results. However, Phan *et al*.’s investigation did not include several key studies pertaining to BPA^8,22–29^ and pulmonary vasodilators^12,33,34,39,43,46^. Wang *et al*. also compared riociguat to BPA in patients with inoperable CTEPH^58^. The results in this investigation matched those in our overall investigation and our sensitivity analysis. We believe that our investigation, through its inclusion of multiple pulmonary vasodilator therapies, is more reflective of real world patterns as a large European registry of expert centers^56^, an international physician survey^59^, and the recent French cohort of BPA patients^30^ suggest that CTEPH patients are routinely treated with different pulmonary vasodilators (riociguat, phosphodiesterase-5 inhibitors, endothelin antagonists, and prostacyclins) and many patients are on combination therapy. Additionally, the Wang investigation did not evaluate safety outcomes for pulmonary vasodilator therapies. Khan *et al*.^60^ and Zoppellaro *et al*.^61^ evaluated the benefit of BPA in patients with inoperable CTEPH through single-arm meta-analyses of the available BPA data. Both investigations demonstrated improvements in mean 6MWD, mPAP and PVR with effect estimates that were similar to ours. However, neither investigation had a comparison with pulmonary vasodilator therapy, thus limiting the generalizability of their study results. Moreover, there was significant duplication of outcome reporting in their investigation from the individual cohorts that were pooled and reported in the Ogawa *et al*. investigation^8^. Nonetheless, our results along with the previously published results show that both BPA and pulmonary vasodilators provide beneficial effects in inoperable CTEPH.

The emergence of pulmonary vasodilator and BPA therapies for inoperable CTEPH reiterates the need for a multi-disciplinary approach to optimize CTEPH treatment. An expert team should collaboratively determine the operability of CTEPH patients and when patients are deemed inoperable, the optimal treatment approach whether BPA, medical therapy, or a combination of both should be determined. In CTEPH centers, the established infrastructure of cardiac catheterization laboratories may facilitate carefully selected specialists to perform BPA to reduce the symptom burden in inoperable CTEPH. However, for patients with operable CTEPH, surgical thromboendarterectomy remains the preferred option and should not be replaced by BPA until head-to-head comparisons can be performed^9^. Additionally, the rise of the hybrid approaches such as combining BPA and medical therapy both pre and post-surgical thromboendarterectomy, may lead to further hemodynamic improvement and improve long-term survival in CTEPH^62^. Aoki *et al*.^63^ and Wiedenroth *et al*.^64^ have previously demonstrated that a combination approach of BPA and pulmonary vasodilators likely exhibits a treatment interaction to improve mPAP, PVR, and WHO FC more than just the isolated use of BPA or pulmonary vasodilators. Our findings also highlight the heterogeneity in the definition of inoperable CTEPH. This is evidenced by the wide variation in functional and hemodynamic status of patients who were deemed inoperable (Table 1). This is also in the context of changing criteria for pulmonary thromboendarterectomy candidacy from primarily anatomic criteria to greater integration of hemodynamics and functional status^7^.

We acknowledge that our investigation has several important limitations. The pooling of data in the form of meta-analyses has well-recognized limitations^65^. Additionally, there are inherent biases in the comparison of observational data for BPA to clinical trial data for medical therapy. We did pool all medical therapy together, but there is evidence in pulmonary arterial hypertension that prostacyclin has greater hemodynamic benefits than oral vasodilators^66^. Moreover, only riociguat is approved for treatment of CTEPH so other medical therapies are considered experimental^9^. However, our investigation was meant to provide a broad overview of the treatment approaches to identify major trends. The greatest limitation is the lack of head-to-head data comparing pulmonary vasodilators and BPA. Our hope is that two ongoing randomized trials, UMIN000019549^67^ and NCT02634203^68^, will provide estimates of each approach’s relative efficacy.

There are important considerations and limitations in our study regarding the effectiveness of BPA. First, there is regional variability in the definition of operable CTEPH. Many investigations in our meta-analyses did not outline the adjudication process for inoperability, and thus the treatment effect of BPA may be exaggerated. Another important consideration for BPA is the emerging experience and continued refinement of the procedure, which may lead to heterogeneity in BPA data. For instance, there is no clear standard on how many procedures of BPA should be done per patient, what the timing between procedures should be, what the optimal technical approach is, and which pulmonary artery segments should be intervened on first^6^. There has been a reduction in the incidence of reported complications since Feinstein first reported the use of BPA for inoperable CTEPH. This was noted in later published series and is likely attributed to improvement in procedural techniques, devices, and greater use of intravascular imaging. The lack of an accepted protocol or standard of when to perform BPA and/or administer pulmonary vasodilator therapies may introduce bias. Furthermore, there may also be treatment interactions between the relief of macrovascular obstruction by BPA and concomitant/subsequent administration of pulmonary vasodilators that may overestimate the treatment benefit of BPA. This hybrid approach requires further investigation before widespread implementation as prior estimates of the costs of pulmonary vasodilator therapy in CTEPH have ranged from $12,000 to $98,000 American dollars per annum^69^. We were unable to fully identify the benefit of hybrid therapy via sensitivity analyses since we did not plan them a priori.

Finally, there were minimal changes in cardiac index in both arms. This was likely biased by limited reporting of cardiac index in the included studies.

## Conclusions

In summary, BPA and pulmonary vasodilators both improve functional and hemodynamic outcomes in patients with inoperable CTEPH. While BPA may offer greater functional and hemodynamic improvements, this technique carries the accompanying risks of an invasive procedure. More high-quality randomized data with long-term follow-up is needed to definitively examine the role of BPA and pulmonary vasodilators for the treatment of inoperable CTEPH.

## Supplementary information

Supplementary information.
