# Supplementary material for: Comparison of Balloon Pulmonary Angioplasty and Pulmonary Vasodilators for Inoperable Chronic Thromboembolic Pulmonary Hypertension: A Systematic Review and Meta-Analysis
Source: Sci Rep. 2020 Jun 1;10:8870. doi: 10.1038/s41598-020-65697-4 (PMC7264327; doi:10.1038/s41598-020-65697-4)

Supplemental Appendices

Comparison of Balloon Pulmonary Angioplasty and Pulmonary Vasodilators for Inoperable Chronic Thromboembolic Pulmonary Hypertension: A Systematic Review and Meta-Analysis

---

Rajat Kalra, MBChB, Sue Duval, PhD, Thenappan Thenappan, MD, Ganesh Raveendran, MD, MS, Marc Pritzker, MD, Sasha Z. Prisco, MD, PhD, and Kurt W. Prins, MD, PhD

Affiliations:
1. Cardiovascular Division, University of Minnesota, Minneapolis, MN, U.S.A.

Supplemental Appendix 1: Study Protocol

Supplemental Appendix 2: Detailed Search Strategy

Supplemental Appendix 3: Definitions of Outcomes

Supplemental Appendix 4: Method of Transforming Study-Level Data to Consistent Forms

Supplemental Appendix 5: MOOSE Checklist for Meta-Analyses of Observational Studies

Supplemental Appendix 6: Study Quality Assessment

Supplemental Figure 1: Funnel Plot to Evaluate Small Study Effects in Balloon Pulmonary Angioplasty Arm

Supplemental Figure 2: Funnel Plot to Evaluate Small Study Effects in Pulmonary Vasodilator Arm

Supplemental Appendix 1: Study Protocol

Study Protocol
Methods for the literature search, data extraction, and analysis were specified in advance as outlined below:

Inclusion Criteria
Studies meeting all of the following criteria will be included:
1. Published studies will be included regardless of date of publication.
2. Manuscript must be available in the English language.
3. Human subjects.
4. Studies with greater than or equal to five patients.
5. Among investigations evaluating the same cohort, we will take the series that reports the outcomes on the largest cohort with the most complete outcome reporting.

Exclusion Criteria
Studies will be excluded if any of the following criteria are met:
1. Non-English manuscripts.
2. Studies in animals, imaging studies.
3. Studies that fail to report the primary outcome (change in six-minute walk distance).
4. Studies that report outcomes for a cohort of patients whose outcomes have already been reported.

Systematic Literature Search
An electronic search of SCOPUS will be performed for relevant published observational data and randomized clinical trials. References of identified studies will also manually be searched for relevant publications. The search will be independently implemented by two study investigators (R.K., K.W.P.). The detailed search strategies for balloon pulmonary angioplasty and for the individual medical therapies is outlined in **Supplemental Appendix 2**.

Data Selection and Endpoints
The primary objective of this analysis is to evaluate the impact that balloon pulmonary angioplasty (BPA) and medical therapies have on six-minute walk distance in patients with inoperable chronic thromboembolic pulmonary hypertension or chronic thromboembolic pulmonary hypertension that is persistent or recurrent after surgery. Data extracted from each study will include: 1) study details, including year, location, country, number of patients, follow-up duration, age of patients, proportion of male patients, functional status, baseline pulmonary vasodilator usage, baseline anticoagulation usage, pre-therapy hemodynamics, functional status after intervention, hemodynamics after intervention and 2) safety outcomes, including, all-cause mortality, wire injuries (BPA arm), and serious adverse events. For studies pertaining to medical therapy, we will also collect data on the following outcomes: type of medical therapy, route, protocol, mean/median dose, and adverse effects. For studies evaluating BPA, we will collect additional data on the following outcomes: procedure protocol, number of catheterizations, number of vessels intervened upon, and procedure-related adverse outcomes. The definitions for these are presented below in **Supplemental Appendix 3.** A single investigator will perform data extraction (R.K.) with random and blinded verification for consistency in data extraction by two other authors (K.W.P. and T.T.). All discrepancies in data extraction will be resolved by mutual consensus.

Statistical Analysis
Meta-analysis of both continuous variables and proportions will be done with random effect modeling to produce the most conservative effect estimates. A continuity correction of 0.5 will be used in meta-analysis of proportions. Sensitivity analyses will be done for the primary outcome if heterogeneity exceeds 50%. The REML method will be used for meta-regression of study-level covariates to explore heterogeneity in outcomes.

We will use the Trim and Fill method to assess for small study effects if the number of studies in the arm exceeds 10. Egger's regression will also be used to evaluate for small study effects.

Sensitivity Analyses

We will compare change in 6MWT between the different pulmonary vasodilators. We will also evaluate whether differing procedural volumes are associated with different change in 6MWT outcomes. Riociguat will also be evaluated in isolation against the BPA therapies since it is the only FDA-approved pulmonary vasodilator.

Supplemental Appendix 2: Detailed Search Strategies

The search strategy will be used in the SCOPUS database was searched from 1945 till December 2018 for eligible studies using a prespecified term list. SCOPUS catalogues MEDLINE, Embase, Compendex, the World Textile index, Fluidex, Geobase, and Biobase. If there are studies of interest that the search yields and we are unable to access them, we will seek them via personal contact with study authors. The following search strategy were employed for balloon pulmonary angioplasty:

'Chronic thromboembolic pulmonary hypertension angioplasty'
TITLE-ABS-KEY (chronic AND thromboembolic AND pulmonary AND hypertension AND angioplasty)

Individual searches were done for each medical therapy that we investigated:

'Chronic thromboembolic pulmonary hypertension ambrisentan'
TITLE-ABS-KEY (chronic AND thromboembolic AND pulmonary AND hypertension AND ambrisentan)

'Chronic thromboembolic pulmonary hypertension beraprost'
TITLE-ABS-KEY (chronic AND thromboembolic AND pulmonary AND hypertension AND beraprost)

'Chronic thromboembolic pulmonary hypertension bosentan'
TITLE-ABS-KEY (chronic AND thromboembolic AND pulmonary AND hypertension AND bosentan)

'Chronic thromboembolic pulmonary hypertension epoprostenol'
TITLE-ABS-KEY (chronic AND thromboembolic AND pulmonary AND hypertension AND epoprostenol)

'Chronic thromboembolic pulmonary hypertension macitentan'
TITLE-ABS-KEY (chronic AND thromboembolic AND pulmonary AND hypertension AND macitentan)

'Chronic thromboembolic pulmonary hypertension riociguat'
TITLE-ABS-KEY (chronic AND thromboembolic AND pulmonary AND hypertension AND riociguat)

'Chronic thromboembolic pulmonary hypertension selexipag'
TITLE-ABS-KEY (chronic AND thromboembolic AND pulmonary AND hypertension AND selexipag)

'Chronic thromboembolic pulmonary hypertension sildenafil'
TITLE-ABS-KEY (chronic AND thromboembolic AND pulmonary AND hypertension AND sildenafil)

'Chronic thromboembolic pulmonary hypertension tadalafil'
TITLE-ABS-KEY (chronic AND thromboembolic AND pulmonary AND hypertension AND tadalafil)

'Chronic thromboembolic pulmonary hypertension treprostinil'
TITLE-ABS-KEY (chronic AND thromboembolic AND pulmonary AND hypertension AND treprostinil)

Supplemental Appendix 3: Definitions of Outcomes

In all instances, the authors' definition of the below mentioned characteristics was used. Where the definition has evolved since the time of publication or there were multiple interpretations, we used the following definitions:

Follow-up	Follow-up was measured in person years. This number was derived by multiplying the mean or median follow-up in months (as stated by authors) by the number of patients and then dividing by 12. If the authors did not state follow-up, survival to discharge was the measured follow-up that we reported.

Procedural Characteristics

Number of catheterizations	This was defined as the number of dilation sessions that the patient underwent.
Number of vessels treated	This was defined as the number of individual vessels that were intervened upon during the catheterizations.

Safety Outcomes

All-cause mortality	This was defined as mortality at the end of follow-up period, after the patient received the intervention.
Significant adverse events	This included any adverse event that led the patient to be withdrawn from the trial, to be hospitalized, or any event that was classed as 'significant' by the authors.
Wire Injuries	This category included the composite outcome of vessel dissection, vessel perforation, or anything classed by the authors as a 'wire injury'.
Reperfusion edema	This category included any report of reperfusion edema by the authors.

Supplemental Appendix 4: Method of Transforming Study-Level Data to Consistent Forms

Studies reported results in several different ways.

1. Mean and standard deviation (SD) for each of treatment and control, along with an exact p-value. These SDs are not useful in calculating a treatment effect (difference between pre-treatment and post-treatment), since they do not take the correlation within subjects into account. In this case, the p-value was used to back-calculate the corresponding t-value from a paired t-test, and from that, the appropriate standard error of the difference between pre-treatment and post-treatment conditions was generated.

2. Mean and standard deviation (SD) for each of treatment and control, along with a p-value expressed as > or < some value (usually 0.05). Again, the SDs are not useful in calculating a treatment effect (difference between pre-treatment and post-treatment), since they do not take the correlation within subjects into account. In this case, the p-value was used to back-calculate the corresponding t-value, and from that, the appropriate standard error of the difference between pre-treatment and post-treatment conditions. Where the p-value was given as $p<0.05$, a value of 0.049 was used. Where the p-value was given as $p>0.05$, a value of 0.1 was used. The choice of 0.049 and 0.1 are arbitrary.

3. Median and IQR (25th, 75th percentile) for each pre-treatment and post-treatment. Studies likely reported medians as the data were skewed; however medians and means cannot be combined in a meta-analysis. Hence, medians were converted to means using the formula: (q1+median+q3)/3, where q1=25th percentile and q3=75th percentile. The SD was approximated by (q3-q1)/1.35.

4. Mean and standard deviation (SD) for each of treatment and control. To calculate the SD of the treatment effect, an assumption regarding the within-subject correlation must be made. Using a correlation of 0.05, the following formula was used to calculate the SD of the treatment effect: $S_{diff} = \sqrt{S_1^2 + S_2^2 - 2 \times r \times S_1 \times S_2}$

5. Median and IQR of the difference between treatment and control, and p-value of difference. Medians converted to mean as in (4) above. The p-value was used to back-calculate the corresponding t-value, and from that, the appropriate standard error of the difference between pre-treatment and post-treatment conditions.

6. Where one-sided p-values were reported, they were converted to two-sided to allow combination with the rest of the studies.

7. In those cases where paired differences and their variability were reported, these were used directly in the analyses.

8. If CI reported, SE=(upper limit-lower limit)/3.92
9. If SD reported, SE=SD/sqrt(n)
10. SD_{diff} = sqrt(SD_{pre}^2 + SD_{post}^2)

Supplemental Appendix 5: MOOSE Checklist for Meta-analyses of Observational Studies

Item No	Recommendation	Reported on Page No
Reporting of background should include		
1	Problem definition	3-5
2	Hypothesis statement	5
3	Description of study outcome(s)	6-7, Supplemental Appendix 3
4	Type of exposure or intervention used	6-8
5	Type of study designs used	6-8
6	Study population	6-8
Reporting of search strategy should include		
7	Qualifications of searchers (eg, librarians and investigators)	Title Page
8	Search strategy, including time period included in the synthesis and key words	6, Supplemental Appendix 2
9	Effort to include all available studies, including contact with authors	6, Supplemental Appendix 2
10	Databases and registries searched	6, Supplemental Appendix 2
11	Search software used, name and version, including special features used (eg, explosion)	NA
12	Use of hand searching (eg, reference lists of obtained articles)	Figure 1
13	List of citations located and those excluded, including justification	6, 9, 18-22, Figure 1
14	Method of addressing articles published in languages other than English	6
15	Method of handling abstracts and unpublished studies	6
16	Description of any contact with authors	NA
Reporting of methods should include		
17	Description of relevance or appropriateness of studies assembled for assessing the hypothesis to be tested	6-8, Supplemental Appendix 1
18	Rationale for the selection and coding of data (eg, sound clinical principles or convenience)	6-8, Supplemental Appendix 1
19	Documentation of how data were classified and coded (eg, multiple raters, blinding and interrater reliability)	6-8, Supplemental Appendix 1
20	Assessment of confounding (eg, comparability of cases and controls in studies where appropriate)	6-8, Supplemental Appendix 1

21	Assessment of study quality, including blinding of quality assessors, stratification or regression on possible predictors of study results	8-9, Supplemental Appendix 6
22	Assessment of heterogeneity	9-12, Tables 1-2, Figures 2-3
23	Description of statistical methods (eg, complete description of fixed or random effects models, justification of whether the chosen models account for predictors of study results, dose-response models, or cumulative meta-analysis) in sufficient detail to be replicated	6-8, Supplemental Appendix 1, Supplemental Appendix 6
24	Provision of appropriate tables and graphics	Tables 1-2, Figures 1-3
Reporting of results should include		
25	Graphic summarizing individual study estimates and overall estimate	Figures 2-3
26	Table giving descriptive information for each study included	Tables 1-2
27	Results of sensitivity testing (eg, subgroup analysis)	11-12
28	Indication of statistical uncertainty of findings	9-17

Item No	Recommendation	Reported on Page No
Reporting of discussion should include		
29	Quantitative assessment of bias (eg, publication bias)	Supplemental Figure 1
30	Justification for exclusion (eg, exclusion of non-English language citations)	Figure 1
31	Assessment of quality of included studies	Supplemental Appendix 6
Reporting of conclusions should include		
32	Consideration of alternative explanations for observed results	9-13
33	Generalization of the conclusions (ie, appropriate for the data presented and within the domain of the literature review)	9-14
34	Guidelines for future research	13-14
35	Disclosure of funding source	Title Page

From: Stroup DF, Berlin JA, Morton SC, et al, for the Meta-analysis Of Observational Studies in Epidemiology (MOOSE) Group. Meta-analysis of Observational Studies in Epidemiology. A Proposal for Reporting. *JAMA*. 2000;283(15):2008-2012. doi: 10.1001/jama.283.15.2008.

Supplemental Appendix 6: Study Quality Assessment with the Newcastle-Ottawa Scale

Selection of Exposed and Non-Exposed Cohorts					Comparability	Outcome of Interest		
**Study Name**	**Representativeness**	**Selection of Non-Exposed Cohort**	**Ascertainment of Exposure**	**Demonstration that Outcome Was Not Present at Start of Study**	**Comparability of Cohorts**	**Assessment of Outcome**	**Length of Follow-up**	**Adequacy of Follow-up**
**Balloon Pulmonary Angioplasty**								
Feinstein *et al*, 2001	*	NR	*	*	NR	*	*	*
Roik *et al*, 2016	*	NR	*	*	NR	*	*	*
Moriyama *et al,* 2017	*	NR	*	*	NR	*	*	*
Ogawa *et al*, 2017	*	NR	*	*	NR	*	*	NR
Yamasaki *et al*, 2017	*	NR	*	*	NR	*	*	*
Kriechbaum *et al*, 2018	*	NR	*	*	NR	*	*	*
Kurzyna *et al*, 2018	*	NR	*	*	NR	*	*	*
Kwon *et al*, 2018	*	NR	*	*	NR	*	*	*
Velazquez *et al*, 2018	*	NR	*	*	NR	*	*	*
Yamagata *et al*, 2018	*	NR	*	*	NR	*	*	*
Brenot *et al*, 2019	*	NR	*	*	NR	*	*	*
**Pulmonary Vasodilators**								
Ghofrani *et al*, 2003	*	NR	*	*	NR	*	*	*
Scelsi *et al*, 2004	*	NR	*	*	NR	*	*	*
Bonderman *et al*, 2005	*	NR	*	*	NR	*	*	*
Hoeper *et al*, 2005	*	NR	*	*	NR	*	*	*
Hughes *et al*, 2006	*	NR	*	*	NR	*	*	*
Cabrol *et al*, 2007	*	NR	*	*	NR	*	*	NR
Reichenberger *et al*, 2007	*	NR	*	*	NR	*	*	*
Segovia Cubero *et al*, 2007	*	NR	*	*	NR	*	*	*
Seyfarth *et al*, 2007	*	NR	*	*	NR	*	*	*
Skoro-Sajer *et al*, 2007	*	*	*	*	NR	*	*	*
Rossi *et al*, 2008	*	NR	*	*	NR	*	*	*
Post *et al*, 2009	*	NR	*	*	NR	*	*	NR
Vassallo *et al*, 2009	*	NR	*	*	NR	*	*	*
Ghofrani *et al*, 2010	*	*	*	*	NR	*	*	*
Yamamoto *et al*, 2019	*	NR	*	*	NR	*	*	-
Van Thor *et al*, 2019	*	NR	*	*	NR	*	*	*
**Case-Control Studies**								
**Selection**					**Comparability**	**Exposure**		

Study Name	Adequacy of Case Definition	Representativeness	Selection of Controls	Definition of Controls	Comparability	Ascertainment of Exposure	Same Method of Ascertainment for Cases and Controls	Non-Response Rate
Vizza *et al*, 2006	NR	*	*	NR	*	*	NR	NR

Legend: *=fulfills some criteria for reporting; **=fulfills all criteria for reporting; NA=not applicable; NR=not reported.

Study ID	Experimental	Comparator	Randomization process	Deviations from intended interventions	Missing outcome data	Measurement of the outcome	Selection of the reported result	Overall
Jais 2008	Bosentan	Placebo	+	+	+	+	+	+
Suntharalingam 2008	Sildenafil	Placebo	+	?	+	+	+	?
Ghofrani 2013	Riociguat	Placebo	+	+	+	+	+	+
Ghofrani 2017	Macitentan	Placebo	+	+	+	+	+	+
Sadaushi-Kolici 2018	Treprostinil (high)	Treprostinil (low)	+	+	+	+	+	+
Escribano-Subias 2019	Ambrisentan	Placebo	+	+	+	+	+	+

Supplemental Figure 1: Funnel Plot to Evaluate Small Study Effects in Balloon Pulmonary Angioplasty Arm

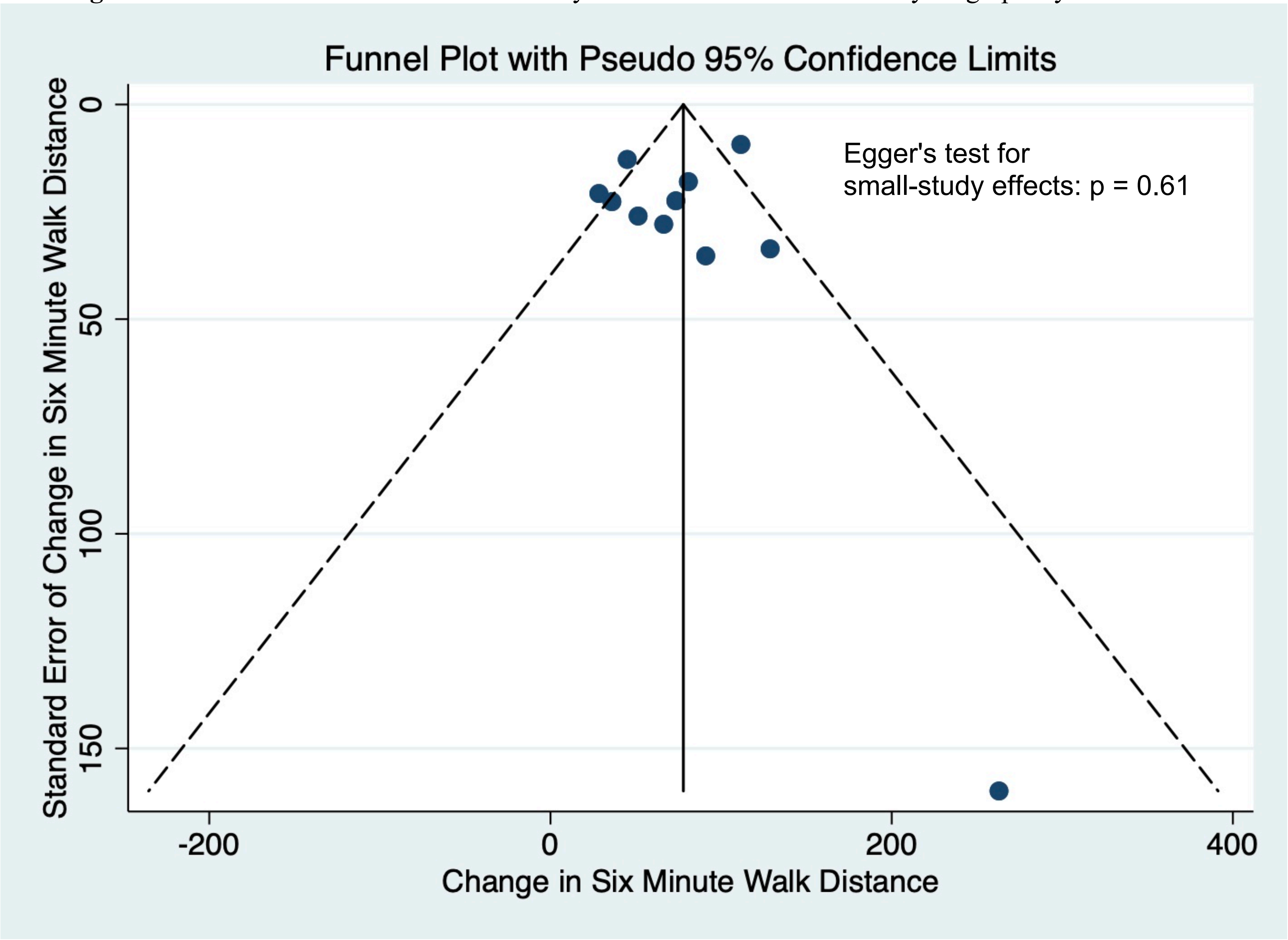

Supplemental Figure 2: Funnel Plot to Evaluate Small Study Effects in Pulmonary Vasodilator Arm

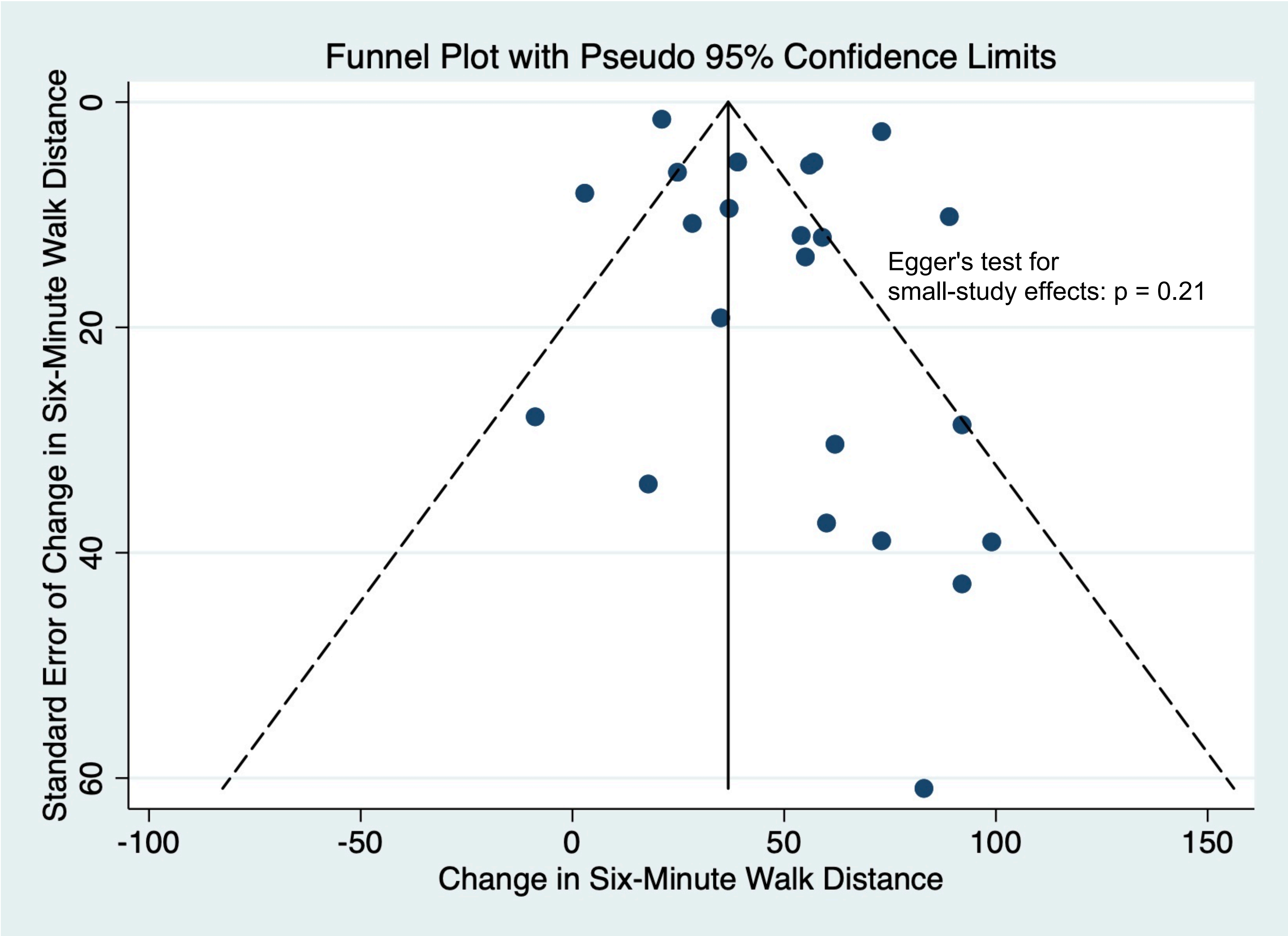

Supplement: Supplementary file 1 — Supplementary information. [file 41598_2020_65697_MOESM1_ESM.pdf]
